# A Case Treated with the Tissue Remedies

**Published:** 1889-01

**Authors:** E. H. Holbrook

**Affiliations:** Baltimore, Md.


### A CASE TREATED WITH THE TISSUE REMEDIES.

E. H. Holbrook, M. D., Baltimore, Md.

November 21st, 1888, I was called to see Miss B. J., a young lady about sixteen or eighteen years of age. Her mother told me she was taken sick on the 16th with severe pains in the knees, which changed to the heel, and from there to the region of the heart. These changes had been caused by the application of some proprietary medicine with which she bathed the parts. For a few days previous to her sickness she was perceived by her mother to be very languid, and would stop her sewing and lay her head down on her arm and go to sleep. Together with the pains there were profuse hemorrhages from the nose.

Her mother, failing in her treatment, and becoming alarmed at the unpleasant symptoms which had become developed, called in a physician in the neighborhood, who prescribed Comp. Licorice Mixture, with the addition of Paregoric, to be given in tablespoonful doses every three hours. After a few days' treatment and no relief being obtained from his medication, the young lady told the physician she would not take his medicine any longer, and dismissed him. I saw her between three and four P. M. on the 21st, and found her suffering very much from an agonizing pain in the region of the heart, which was beating as if it was trying to force itself through the walls of the thorax. She also labored greatly in her breathing, complaining of a smothered feeling. Tongue was coated dark-brown, and as dry as a chip; tenderness in ileo-cœcal region, pulse full and strong, high fever, and when she falls asleep she begins a rambling talk, which continues, especially throughout the night.

I gave at this visit *Fer. phos.*$^{200}$ in water, a teaspoonful every hour. Between eight and nine P. M. I saw her again. The pulse had diminished considerably, and the face and forehead were covered with perspiration. But she said the pain about the heart was worse. I now stopped the *Fer. phos.* and gave *Kali phos.*$^{cm}$ (Swan) in water, a teaspoonful every hour, except when sleeping quietly. The next morning I found her sitting up in bed reading a book, greatly relieved in every way, except that a diarrhœa had set in. Thinking the remedy might stop that, I continued it that day, but found it had not the next morning. After examining the movement and questioning the mother, I found she was not disturbed through the night, but had her first movement about four A. M. It was green and of a granular nature. I now gave *Nat. sulph.*$^{cm}$ (Swan) in water, a teaspoonful every hour until diarrhœa is better, then every two hours. The following morning I found the diarrhœa checked, tongue becoming moist and cleaning, and the young lady in fine spirits. The diarrhœa stopped soon after commencing the last remedy. From this time there was a steady improvement, and on Monday, the 26th, she was able to sit up, all soreness in the bowels was gone, and she had a desire for food. (Was this a case of typhoid fever thus cut short, or what might it be *called?*)

